# [Retracted] Slit2 suppresses endothelial cell proliferation and migration by inhibiting the VEGF-Notch signaling pathway

**DOI:** 10.3892/mmr.2025.13572

**Published:** 2025-05-20

**Authors:** Guo-Jian Li, Yong Yang, Guo-Kai Yang, Jia Wan, Dao-Lei Cui, Zhen-Huan Ma, Ling-Juan Du, Gui-Min Zhang

Mol Med Rep 15: 1981–1988, 2017; DOI: 10.3892/mmr.2017.6240

Following the publication of the above paper, it was drawn to the Editor's attention by a concerned reader that the Transwell migration assay data shown in Fig. 2 on p. 1984 had already appeared in an article written by different authors at different research institutes that was published previously in the journal *Drug Design, Development and Therapy*.

Owing to the fact that the contentious data in the above article had already been published prior to its submission to *Molecular Medicine Reports*, the Editor has decided that this paper should be retracted from the Journal. After having been in contact with the authors, they accepted the decision to retract the paper. The Editor apologizes to the readership for any inconvenience caused.

